# Danish general practice under threat?

**DOI:** 10.1080/02813432.2019.1684431

**Published:** 2019-11-18

**Authors:** Jørgen Nexøe

**Affiliations:** Danish National Editor, Editorial Board Scandinavian Journal of Primary Health Care; Research Unit of General Practice, University of Southern Denmark

General practice in Denmark is characterized by five core components: a list system, with an average of about 1600 persons on the list of a typical general practitioner; the general practitioner as gatekeeper and first-line provider in the sense that a referral from a general practitioner is required for most office-based specialists and always for in- and outpatient hospital treatment; an after-hours system staffed by general practitioners on a rota basis; a mixed capitation and fee-for-service system; and general practitioners are self-employed, working on contract for the public funder based on a national agreement [1].

The number of general practitioners is decreasing. The lack of general practitioners is worst in Region North Jutland and Region Zealand but has gradually spread to very large parts of the country including the Capital Region. This poses major challenges for the primary health care system. Because at the same time the number of patients increases, the Danes live longer, and more patients get to retirement age. There is also an increase in the number of citizens with chronic disorders, both because the population get more elderly, and new treatment options mean that many live for longer periods with one or more chronical diseases.

An increasing proportion of the population therefore needs to see their general practitioner frequently, which increases the pressure on the fewer remaining general practitioners who are increasingly handling more and more patients requiring treatment.

In many places in the country, patients find that when their doctor go on retirement, new doctors are not ready to take over. Today, there are about 127,000 citizens who are not enlisted with a self-employed general practitioner, but rather receive primary health care from a clinic run by a private company or a temporary clinic run by one of the administrative regions.

In a few years, this number will probably have increased to almost 300,000 Danes not enlisted with a self-employed general practitioner.

The lack of general practitioners is partly due to a general lack of doctors, but also that more doctors trained in general practice choose other employment rather than becoming a general practitioner. About 770 specialist doctors trained in general medicine that are not working in general practice, have found employment in other fields including emergency and psychiatric hospital departments.

The traffic of specialist doctors in general practice to other specialties may depend on several factors. There may be specialists in general practice who are at the beginning of their careers and have not yet found the practice that will form the basis of their future in general practice. There may also be some specialists in general practice who find pay and working conditions as a public servant more attractive than the job as self-employed general practitioner.

In a Nordic analysis by QUALICOPC [2], the number of face-to-face patient contacts is estimated to 24 in clinics in Denmark 2014, which is somewhat higher than what you see in the other Nordic countries. The fact that patient contacts face-to-face in the clinic is significantly lower in the other Nordic countries may be because in Finland, Norway and Sweden user fees are paid for doctor visits, which can help to dampen the demand and ease the pressure on the general practitioners. In the same analysis, it is seen that the general practitioners in Denmark have the highest weekly number of hours of direct patient treatment compared to the other Nordic countries.

In 2012 and 2016, the Research Unit for General Practice in Aarhus conducted surveys [3] among the general practitioners and in just four years there has been a marked increase in the number of general practitioners who find that their work has become more demanding. In 2012, approx. 25% of the doctors agreed that they were somewhat or much bothered that their work had become more demanding. This figure more than doubled to 58% in 2016.

Despite these facts, a vast majority in the Danish parliament have decided that more chronically ill patients today taken care of in hospital outpatient clinics, in the future must be treated by general practitioners. This requires the number of general practitioners to increase. The Organisation of General Practitioners has estimated that about 5000 general practitioners will be needed in 2030 – almost 1700 more than today. This may be attained by educating more doctors in the universities. Recently the Danish parliament have decided to increase admission to medical school and to establish two new educational facilities and consequently, the goal of 5000 general practitioners may be reached.

Some tasks e.g., selected preventive measures performed by general practitioners today may have to be outsourced to health visitors, midwifes and nurses in the municipal health service.

The current structure and position of general practices have developed over more than 100 years. General practice has provided cost-efficient, first-line services and careful gatekeeping under ever changing circumstances. Within the last few years the Danish general practice system with self-employed GPs may have reached its limit regarding flexibility and the very system may be threatened. Already, since 2014 the primary health care out-of-hours service in the Capital Region has been run by nurses employed by the Region triaging under the supervision of doctors that may or may not be general practitioners also employed by the Region. An increasing number of general practitioners receive a fixed monthly salary and are no longer self-employed.

Only the future will show if measures taken and to be taken will be enough to maintain the unique Danish primary health care system with the general practitioner as a core character.
